# The Influence of Butter and Oils on Oxidative Reactions during In Vitro Gastrointestinal Digestion of Meat and Fish

**DOI:** 10.3390/foods10112832

**Published:** 2021-11-17

**Authors:** Thomas Van Hecke, Stefaan De Smet

**Affiliations:** Laboratory for Animal Nutrition and Animal Product Quality, Department of Animal Sciences and Aquatic Ecology, Ghent University, Coupure Links 653, B-9000 Ghent, Belgium; thomas.vanhecke@ugent.be

**Keywords:** 4-hydroxy-2-hexenal, 4-hydroxy-2-nonenal, protein carbonyl compounds, vegetable oil, fish oil

## Abstract

Oxidative reactions during cooking and gastrointestinal digestion of meat and fish lead to the formation of various lipid- and protein oxidation products, some of which are toxic. In the present study, it was investigated how the addition of 3% butter or oils affect lipid- and protein oxidation during cooking and in vitro digestion of meat (chicken thigh, chicken breast, beef) and fish (mackerel, cod). These muscle foods were selected based on their differences in heme-Fe and PUFA contents, and n-6/n-3 PUFA ratio, and therefore varying potential to form oxidation products during digestion. Without additional fat, mackerel digests displayed the highest n-3 PUFA oxidation (4-hydroxy-2-hexenal, propanal, thiobarbituric reactive acid substances), and chicken digests the highest n-6 PUFA oxidation (4-hydroxy-2-nonenal, hexanal), whereas both lipid- and protein oxidation (protein carbonyl compounds) were low in cod and beef digests. Lipid oxidative reactions were generally not altered by the addition of butter to any muscle matrix, whereas the addition of fish oil and safflower oil in different ratios (3:0, 2:1, 1:2, 0:3) as n-3 PUFA and n-6 PUFA source respectively, stimulated oxidative reactions, especially during digestion of beef. Since beef was considered the muscle matrix with the highest potential to stimulate oxidation in the added fat substrate, in a second experiment, beef was cooked and digested with 3% butter or seven commercial vegetable oils (sunflower-, maize-, peanut-, rapeseed-, olive-, rice bran- or coconut oil), all labeled ‘suitable for heating’. No relevant oxidative reactions were however observed during digestion of beef with any of these commercial vegetable oils.

## 1. Introduction

With the aim to reduce the risk of developing cardiovascular disease, many nutritional and health bodies promote the replacement of dietary saturated fatty acids (SFA) with monounsaturated fatty acids (MUFA) and polyunsaturated fatty acids (PUFA). For example, the Flemish dietary guidelines specifically advise to replace butter as much as possible with vegetable oils for culinary preparations [1]. However, several oils are rich in PUFA which are sensitive to oxidative damage during storage and gastrointestinal digestion. Formed oxidation products may oxidize low density lipoprotein (LDL) upon their absorption in the bloodstream, and ox-LDL is considered a pro-atherosclerotic factor. For instance, the consumption of turkey meat increased circulating ox-LDL in humans, which was related to the oxidation of PUFA during gastrointestinal digestion of the meat [2]. In the latter study, marinating the turkey with red wine reduced lipid oxidation and inhibited an increase of ox-LDL in blood.

Limited information is available how a replacement of butter with PUFA-rich oils for the culinary preparation of muscle foods affect their oxidative stability during gastrointestinal digestion. The extent of oxidative damage to PUFA and protein occurring during gastrointestinal digestion of meat, oils and other foods, depends on the characteristics of the digested meal [3,4]. Among in vitro digested muscle foods originating from 18 different mammal, poultry and fish species, beef contained relatively high amounts of pro-oxidant heme-Fe, but, due to their very low content of oxidizable PUFA, one of the lowest amounts of lipid- and protein oxidation products were found in their digests [5], which agrees with the findings previously reported by Steppeler et al. [6]. However, when beef was digested in combination with n-3 PUFA rich fish oil [6], or lard [5,7], a large increase in oxidative reactions was observed. In fact, most animal feeding studies demonstrating pro-oxidant effects of heme-Fe, blood sausage, or beef simultaneously included n-6 PUFA rich vegetable oils in their diet, such as 2–5% safflower oil [8,9,10,11], 4% corn oil [12] or 13.7–15% sunflower oil [13,14], whereas rats consuming heme-Fe with saturated coconut oil did not demonstrate increased oxidation in their intestines [9]. Likewise, when sunflower oil (13.7%) was added to a diet containing red meat (17.1%), rats had increased plasma levels of n-6 PUFA oxidation products and ox-LDL, along with a worsened endothelial dysfunction and atherosclerosis compared to rats on the experimental meat diet without added sunflower oil [14]. Besides, also MUFA may impact on the oxidative stability since oleic acid in olive oil decreased oxidation during digestion of turkey meat [15], however pro-oxidant effects of extra-virgin olive oil during turkey meat digestion have been observed as well [16]. Various reactive lipid oxidation products such as 4-hydroxy-2-hexenal (4-HHE) and 4-hydroxy-2-nonenal (4-HNE) are able to carbonylate protein, which is one of the pathways to stimulate protein oxidation [17].

In previous research, the fatty acid composition was the most influential factor determining the lipid- and protein oxidation profile in in vitro gastrointestinal digests of a wide range of cooked muscle foods [5]. Their n-3 PUFA content correlated well with the levels of their specific oxidation products 4-HHE and propanal, but also with the content of thiobarbituric acid reactive substances (TBARS) in digests, whereas the levels of the n-6 PUFA specific oxidation products 4-HNE and hexanal in digests were associated with the muscular n-6/n-3 PUFA ratio, but not with their absolute n-6 PUFA content. Therefore, we previously hypothesized that n-3 PUFA could possibly protect n-6 PUFA from oxidation by mechanisms of ‘sacrificial oxidation’. A similar mechanism is described in protein where the oxidation of methionine protects other amino acids from oxidation [18].

Present research aimed to elucidate how a replacement of butter with oils affects the oxidative stability of muscle foods during cooking and gastrointestinal digestion, considering the nature of the used cooking fat source (fatty acid profile), and the muscle food (fatty acid content and profile, heme-Fe). In a first experiment, different proportions of fish oil (n-3 PUFA source) and safflower oil (n-6 PUFA source), or butter (low in PUFA) were added to different muscle matrices (mackerel, cod, chicken breast or thigh, beef). These muscles displayed a wide range in fat content and fatty acid profile, and therefore their n-6/n-3 PUFA ratio was either or not sensitive to be altered by the added fat source. In a second experiment, beef was cooked with butter or a range of commercial vegetable oils (sunflower-, maize-, peanut-, rapeseed-, olive-, coconut- and rice bran oil), all labeled as ‘suitable for cooking’. These fat sources are either discouraged (butter, coconut oil) or promoted (other vegetable oils) for the culinary preparation of meals, according to the Flemish dietary guidelines [1].

## 2. Materials and Methods

### 2.1. Chemicals

All digestive enzymes [α-amylase from hog pancreas (~50 U/mg; 10080), mucin from porcine stomach type II (M2378), pepsin from porcine gastric mucosa (>250 U/mg solid; P7000; analyzed activity of 860 U/mg [19]), lipase from porcine pancreas type II (10–400 U/mg protein; L3126), pancreatin from porcine pancreas (8 × USP specifications; P7545), porcine bile extract (B8631)], reagents [2-thiobarbituric acid (T5500), 1,3-cyclohexanedione (C101605), 2,4-dinitrophenylhydrazine (D199303)], analytical standards [propanal (64409, purity ≥98%), HNE-DMA (H9538, purity >85%), hexanal (115606, purity ≥98%) and 1,1,3,3-tetramethoxypropane (108383, purity 99%)], were purchased from Merck (Diegem, Belgium). The analytical standard of 4-HHE (purity ≥98%) was purchased from Sanbio B.V. (Uden, Netherlands).

### 2.2. Experimental Design

In the first experiment, muscles were selected with varying PUFA content, n-6/n-3 PUFA ratio, and heme-Fe levels, whereby the n-6/n-3 PUFA ratio of muscles with relatively low PUFA content are expected to be more easily altered by the addition of the oils. For this purpose, samples originating from mackerel (low heme-Fe, high PUFA content, low n-6/n-3 ratio), cod (low heme-Fe, low PUFA content, low n-6/n-3 ratio), chicken thigh (low heme-Fe, high PUFA content, high n-6/n-3 ratio), chicken breast (low heme-Fe, low PUFA content, high n-6/n-3 ratio), and beef (high heme-Fe, low PUFA content) were collected. Muscles were either or not added with 3% butter or 3% oil, which was a combination of fish oil (n-3 PUFA source) and safflower oil (n-6 PUFA source) at ratios of either 3:0, 2:1, 1:2, or 0:3. Following heating, these muscle foods were subjected to an in vitro gastrointestinal digestion model in quadruplicate. Lipid oxidation (4-HHE, propanal, 4-HNE, hexanal, TBARS) and protein oxidation (PCC) were assessed before and after digestion.

In the second experiment, beef was either or not added with 3% commercially available sunflower-, maize-, peanut-, rapeseed-, olive-, rice bran-, or coconut oil, or butter, all labeled as ‘suitable for cooking’. Following heating, these samples were exposed to in vitro digestion, and oxidation was evaluated in a similar way as the first experiment.

### 2.3. Manufacturing of Meat and Fish Samples

Meat and fish were purchased as fresh as possible from a meat and fish retailer. Muscles were manually chopped into cubes of approximately 5–10 cm^3^, and minced in a grinder (Omega T-12) equipped with a 3.5 mm plate. For the first experiment, raw mackerel (filet), cod (filet), chicken (thigh or breast), and beef (breast) were either or not added with 3% butter (Pure Irish butter, unsalted, Kerrygold, Mitchelstown, Ireland), or 3% of a mixture of fish oil (Fish oil from menhaden, F8020-1L, Sigma Aldrich) and safflower oil (Food grade, 0210288890, MP Biomedicals) in ratios of 3:0, 2:1, 1:2 or 0:3. In the second experiment, beef muscle (different batch from the first experiment, but same muscle and comparable fat content) was either or not mixed with 3% of commercially available sunflower- (Everyday), maize- (Everyday), peanut- (Albert Heijn), rapeseed- (Everyday), olive- (mild, Albert Heijn), rice bran- (King), or coconut oil (BioToday), or butter (Pure Irish butter, unsalted, Kerrygold). All commercial oils were labeled ‘suitable for heating’, and no exogenous antioxidants were listed.

Subsequently, all samples were homogenized manually, packed in anaerobic bags (Allibert, Belgium) in equal proportions, and heated in a warm water bath at 70 °C for 70 min. Following the heat treatment, all meat and fish samples were homogenized in three 5 s bursts using a food processor (Moulinex DP700, France), vacuum packed and stored at −80 °C until in vitro digestion and chemical analyses.

### 2.4. Meat and Fish Characteristics

Lipids in cooked meat and fish were extracted using chloroform/methanol (2/1; *v/v*) and subsequently, fatty acids were methylated and analyzed by gas chromatography (HP6890, Brussels, Belgium) [20]. Nonadecanoic acid (C19:0) was used as an internal standard to quantify the fatty acids. Relative proportions of SFA (sum of C12:0, C14:0, C15:0, C16:0, C17:0, C18:0, C20:0 and C22:0), MUFA (sum of C14:1, C16:1, C17:1, c7C16:1, c9C18:1, c11C18:1, transC18:1 and C20:1) long chain (LC) n-3 PUFA (sum of C20:4n-3, C20:5n-3, C22:5n-3 and C22:6n-3), n-3 PUFA (sum of C18:3n-3 and LC n-3 PUFA), LC n-6 PUFA (sum of C20:2n-6, C20:3n-6, C20:4n-6, C22:4n-6, C22:5n-6) and n-6 PUFA (sum of C18:2n-6, C18:3n-6, and LC n-6 PUFA) were calculated. Hematin was determined spectrophotometrically (Genesys 10S UV-Vis spectrophotometer, Madison, WI, USA) following extraction in aceton/H_2_O/HCl (12 M) solution (40/2/1), and converted to heme-Fe using the formula heme-Fe = hematin × atomic weight Fe/molecular weight hematin [21].

### 2.5. In Vitro Gastrointestinal Digestion

In vitro digestion was performed based on the original digestion protocol of Versantvoort et al. [22], which was adapted to study oxidative reactions during passage in the gastrointestinal system according to Van Hecke et al. ([23], normal conditions). The digestions were performed in quadruplicate and per experiment, all samples were digested on the same day with exactly the same digestive juices to exclude any day effect. In brief, 4.5 g of meat or fish were sequentially incubated at 37 °C for 5 min with 6 mL saliva, 2 h with 12 mL gastric juice, and 2 h with 2 mL bicarbonate buffer (1 M, pH 8.0), 12 mL duodenal juice and 6 mL bile juice. Combinations of the muscle foods with digestive juices result in an initial stomach pH of 2.3, which gradually increases after 2 h stomach digestion to pH 3.5, followed by pH 6.5 in the small intestinal compartment. After completion, samples were homogenized with an ultraturrax (9500 rpm) and aliquots were stored at −80 °C for further analysis

### 2.6. Oxidation Products in Meat, Fish and Digests

Total (free + bound) carbonyls were measured spectrophotometrically in meat, fish and their digests as TBARS (thiobarbituric acid reactive substances) at 532 nm following the reaction with 2-thiobarbituric acid in an acid environment after hydrolysis with NaOH, extraction in 1-butanol, and quantification using a standard curve with 1,1,3,3-tetramethoxypropane [24]. Unbound 4-HHE, propanal, 4-HNE and hexanal in meat, fish and digests were measured by HPLC following their derivatization with cyclohexanedione based on the protocol of Holley et al. [25], with adaptations [26], and each compound was quantified with corresponding analytical standards. Concentrations of PCC were determined spectrophotometrically following reaction with 2,4-dinitrophenylhydrazine according to Ganhão et al. [27].

### 2.7. Statistical Analysis

For the digests of experiment 1, a linear model ANOVA procedure (SAS Enterprise Guide 7) was used with the fixed effects of muscle type, added fat substrate and their interaction term, with Tukey-adjusted post hoc tests performed for all pairwise comparisons. For the digests of the second experiment, an ANOVA procedure was used with the effect of added fat substrate with Tukey-adjusted post hoc tests performed for all pairwise comparisons. *p*-values ≤ 0.05 were considered significant. No statistical analysis was performed on the meat samples prior digestion.

## 3. Results

### 3.1. Experiment 1: Heme-Fe and Fatty Acid Composition

As expected, heme-Fe levels were various folds higher in beef (1.78 ± 0.13 mg/100 g) compared to mackerel (0.40 ± 0.01 mg/100 g), cod (not detected), chicken thigh (0.37 ± 0.02 mg/100 g), and chicken breast (0.13 ± 0.01 mg/100 g).

**Figure 1:**
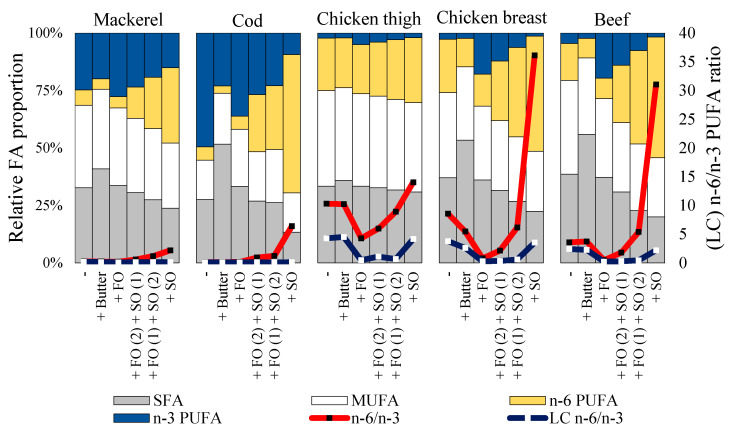
The relative fatty acid profile of cooked muscle foods in combination with 3% butter, or with a mixture of fish oil (FO) and safflower oil (SO) in ratios of either 3:0, 2:1, 1:2, 0:3 (Experiment 1).

The sum of total fatty acids amounted to 2.36 ± 0.04 g/100 g mackerel (from which 0.62 g PUFA), 0.47 ± 0.02 g/100 g cod (0.26 g PUFA), 7.61 ± 0.34 g/100 g chicken thigh (1.90 g PUFA), 1.40 ± 0.04 g/100 g chicken breast (0.36 g PUFA), and 1.40 ± 0.15 g/100 g beef (0.23 g PUFA). Therefore, the PUFA content of mackerel was somewhat lower than intended. Figure 1 shows the relative fatty acid composition of the cooked muscle foods with or without 3% butter or the oil mixtures. Among cooked muscles, fish contained the highest n-3 PUFA proportions and a low n-6/n-3 PUFA ratio (≤0.27), whereas chicken had a relatively high n-6/n-3 PUFA ratio (8.6–10.3). As anticipated in the experimental design, the fatty acid profile of low-fat muscles (cod, chicken breast, beef) were the most sensitive to be altered by the addition of butter and oil mixtures. In low-fat muscles, the addition of butter increased SFA proportions, whereas it largely unaffected the n-6/n-3 PUFA ratio. A relevant decrease in the (LC) n-6/n-3 PUFA ratio was observed when fish oil was added to chicken or beef. Safflower oil largely increased the n-6/n-3 PUFA ratio when added to chicken breast (36:1) and beef (31:1), whereas the LC n-6/n-3 PUFA ratio was unaffected.

### 3.2. Experiment 1: Lipid- and Protein Oxidation

**Figure 2:**
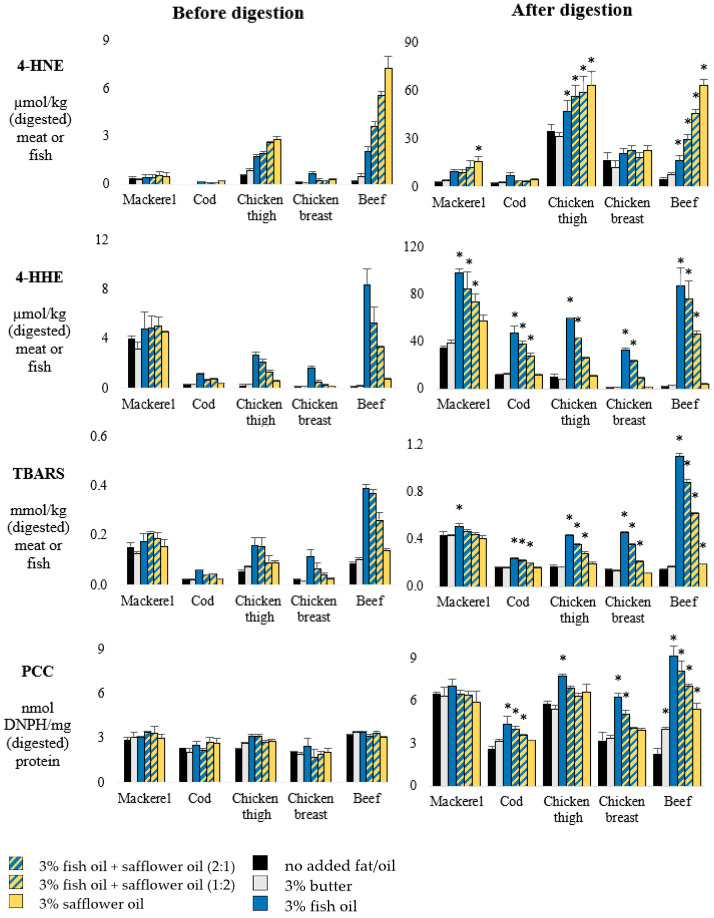
Lipid and protein oxidation in heated muscle-based foods before the addition of digestive juices (**Left**) and after in vitro gastrointestinal digestion (**Right**) (Experiment 1). 4-HNE = 4-hydroxy-2-nonenal, 4-HHE = 4-hydroxy-2-hexenal, TBARS = thiobarbituric acid reactive substances, PCC = protein carbonyl compounds. Error bars indicate standard deviation. Asterisk (*) indicates that the treatment with added fat substrate is significantly different (*p* < 0.05) compared to the muscle food without added fat.

Concentrations of 4-HNE, 4-HHE, TBARS and PCC before and after in vitro gastrointestinal digestion are presented in Figure 2. Patterns of propanal and hexanal reflected the patterns of 4-HHE and 4-HNE respectively, and are therefore not shown. Following gastrointestinal digestion, roughly 10-fold higher levels of 4-HNE and 4-HHE were found, and 2- to 3-fold higher levels of TBARS and PCC compared to the undigested meat and fish. In the absence of an added fat source, among muscle foods, levels of 4-HHE and TBARS were distinctly the highest before and after digestion of mackerel, whereas 4-HNE was particularly present following the digestion of chicken thigh and, to a lower extent, also in chicken breast digests. The addition of butter did not significantly increase any lipid oxidation marker before and after digestion of any muscle type. Among muscle foods, the addition of the oil mixtures had the highest impact on oxidation levels in beef (digests).

In beef (digests), a concentration-dependent significant increase in 4-HNE was observed along with higher added amounts of safflower oil, whereas 4-HHE and TBARS increased along with higher amounts of added fish oil. The two highest levels of added fish oil to beef resulted in similar high levels of TBARS before digestion, and similar 4-HHE levels after digestion. In contrast to the hypothesis, no protective effect of n-3 PUFA rich fish oil on n-6 PUFA oxidation was observed during chicken or beef digestion. Addition of fish oil to fish muscles increased 4-HHE levels in their digests, and also increased 4-HHE and TBARS in both chicken and beef muscles before and after digestion. Next to the previously described pronounced effect of safflower oil on 4-HNE levels in beef (digests), safflower oil also significantly increased 4-HNE in chicken thigh (digests) and to a lower extent in mackerel digests as well.

Whereas before digestion, only subtle differences were present in PCC levels among cooked muscle foods, digests of mackerel, and chicken thigh without added fat source, which contained approximately 2-fold higher PCC levels compared to the digests of cod, chicken breast, and beef. The most pronounced increase of PCC formation during digestion was observed when fish oil was added to beef, resulting in 4-fold higher PCC levels compared to the beef digest without added fat. Fish oil also significantly increased PCC formation during the digestion of chicken breast, chicken thigh, and cod, however to a lesser extent as compared to the effect in beef. Only during beef digestion, PCC formation was also increased by the addition of safflower oil (2.4-fold) and by the addition of butter (+76%).

### 3.3. Experiment 2

**Figure 3:**
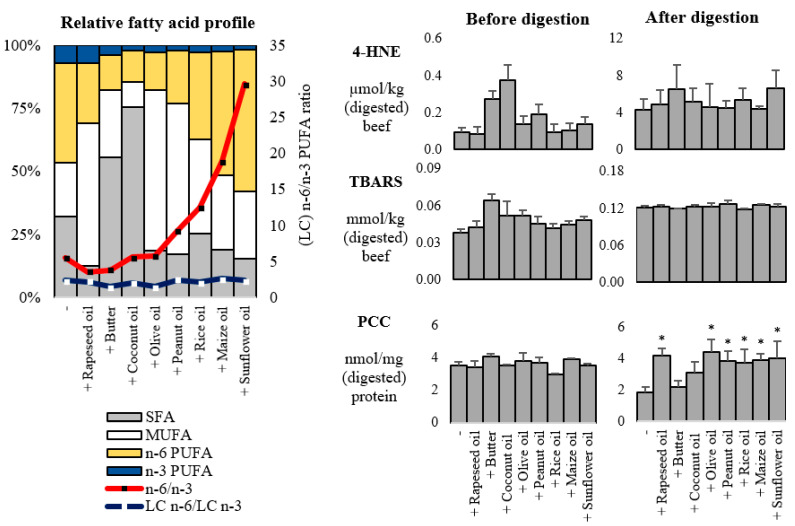
The relative fatty acid profile (**Left**) of beef with or without butter or commercial vegetable oils, and oxidation products (**Right**) 4-HNE (4-hydroxy-2-nonenal), TBARS (thiobarbituric acid reactive substances) and PCC (protein carbonyl compounds) before and after in vitro digestion (Experiment 2). Error bars indicate standard deviation. Asterisk (*) indicates that the treatment with added fat substrate is significantly different (*p* < 0.05) compared to the beef without added fat.

The beef sample used in the second experiment had a total fatty acid content of 0.65 ± 0.12 g/100 g. The relative contribution of MUFA increased 2- to 3-fold with the addition of rapeseed oil, olive oil, peanut oil, and rice oil, whereas the addition of butter and coconut oil more or less doubled relative SFA proportions (Figure 3). Among added fat substrates, the addition of sunflower oil and maize oil increased the n-6/n-3 PUFA ratio the most, up to a ratio of 30 and 19, respectively.

Beef samples with/without butter and their digests had similar concentrations of lipid oxidation products as were found during the first experiment. Beef cooked with butter or coconut oil appeared to have higher levels of 4-HNE and/or TBARS before digestion compared to the other beef samples. However, these higher concentrations were negligible when compared to the levels reached in the first experiment when beef was combined with fish oil or safflower oil. No differences in PCC were observed in the cooked beef samples before digestion. Compared to before digestion, especially beef with/without butter contained lower PCC levels, resulting in significantly higher PCC levels in beef digests including rapeseed-, olive-, peanut-, rice-, maize- and sunflower oil compared to the beef digest without added fat. In contrast to the first experiment, digests of beef with butter contained similar PCC levels as the control beef digests.

## 4. Discussion

In the present study, it was investigated how the replacement of butter with vegetable oils for the culinary preparation of meat and fish, as recommended by e.g., the Flanders Institute for Healthy Living [1], affects the fatty acid profile and oxidative stability of meat and fish during cooking and gastrointestinal digestion. Fish oil is not recommended to be used as a cooking oil, however the microencapsulation of fish oil in meat products is currently being investigated as a strategy to obtain a more desirable fatty acid profile [28,29]. In addition, fish oil was used in the present study to investigate the hypothesis if n-3 PUFA protects n-6 PUFA from oxidation by a mechanism of ‘sacrificial oxidation’, as previously hypothesized [5].

Even though n-6 PUFA are essential nutrients, the typical western diet is reported to contain excessive amounts of n-6 PUFA leading to an unfavorably high n-6/n-3 PUFA ratio of ~15:1, whereas humans evolved on a dietary ratio of 1:1 [30]. In the present study, the replacement of butter with safflower oil largely increased the inherent n-6/n-3 PUFA ratio in lean chicken and beef muscle, up to a ratio of 31:1 to 36:1, whereas the ratio was far less affected when oils were added to PUFA-rich fish or chicken thigh. In agreement, addition of sunflower- or maize oil to beef also increased this ratio to 30:1 and 19:1 respectively in the second experiment. A high ratio has been associated with an increased risk for various diseases such as prostate cancer [31], cardiovascular diseases [30], depression and mood disorders [32,33]. Nevertheless, the physiological relevance of dietary n-6/n-3 PUFA ratios remains controversial since it may merely reflect a deficiency in n-3 PUFA [34,35,36], and the consumption of n-6 PUFA, in particular linoleic acid, may even protect against cardiovascular disease and diabetes type 2 [37,38].

As expected, levels of oxidation products increased various folds during in vitro gastrointestinal digestion, as was previously observed in other digestion studies e.g., [2,5,6,26]. The oxidation profiles in digests of cooked muscles without added fat source were in accordance with previous observations [5,6], showing relatively high n-6 PUFA oxidation (4-HNE, hexanal) in chicken, high n-3 PUFA oxidation (4-HHE, propanal, TBARS) in mackerel, and low oxidation in cod and beef digests. Since butter is predominantly composed of SFA and MUFA, its addition was not accompanied by major changes in the oxidation profile of any cooked muscle. In contrast, the oxidation profile of particularly beef (digests) was largely altered by the added cooking oil mixture, with more n-3 PUFA or n-6 PUFA oxidation along with a larger added proportion of fish oil or safflower oil respectively. Since beef has high contents of the pro-oxidant heme-Fe and inherently low PUFA levels, the nature of the added fat source will largely determine the extent and type of oxidative reactions during digestion of beef. Increased n-3 PUFA oxidation was also observed when beef was in vitro digested in combination with n-3 PUFA rich fish oil [6], and increased n-6 PUFA oxidation when beef was digested with lard [5,7]. Previously, n-6 PUFA oxidation during digestion of 18 different animal muscle species was associated with the n-6/n-3 PUFA ratio, but not with the n-6 PUFA content [5]. This previously led to our hypothesis that n-3 PUFA may be the preferential substrate for oxidation, hereby protecting n-6 PUFA from oxidation, similar to described mechanisms where oxidation of methionine protects other amino acids from oxidation [18]. In contrast to the hypothesis, the addition of n-3 PUFA rich fish oil did not protect n-6 PUFA from oxidation.

The higher formation of PCC during digestion of muscles in the presence of fish- and/or safflower oil is likely the result of more intense carbonylation of protein by higher levels of reactive 4-HNE and 4-HHE, in agreement with previous observations [5]. The addition of oils to mackerel was not accompanied by increased PCC formation during digestion, possibly related to the already relatively high PCC levels in mackerel digests without added fat. Despite absent effects of butter on lipid oxidation, PCC levels were significantly increased when butter was present in beef digests. Kawai et al. [39] reported covalent binding of oxidized cholesteryl esters to protein, and suggested this process is similar to the formation of PCC. Therefore, it could be hypothesized that products arising during the oxidation of cholesterol in butter during beef digestion may contribute to the formation of PCC as well. This finding was however not repeated in the second experiment, using another batch of beef and butter. The reason for this discrepancy is unclear, but could be due to different levels of intrinsic precursors (e.g., cholesterol, vitamin E, etc.) in beef and/or butter.

When beef muscle was cooked with commercial vegetable oils labeled as ‘suitable for heating’, unexpectedly, no increased lipid oxidative reactions were observed during cooking or digestion. Both fish oil and safflower oil from the first experiment, as the commercial oils from the second experiment underwent refining, bleaching and deodorization to increase their stability. Many variations however exist in the processing steps of vegetable oils and accompanying oxidative stability, possibly leading to contrasting results in both experiments. In addition, although not labeled, the addition of antioxidants to increase the oxidative stability of the commercial oils cannot be excluded. It is indeed common practice to enrich commercially available vegetable oils with tocopherols, which are naturally present in the oils, whereas this was not the case with the safflower oil from MP Biomedicals used in the first experiment. During accelerated storage conditions, in which commercial vegetable oils were heated at 70 °C, alkanals and 4-hydroxy-2-alkenals only appeared after circa 5 days for soybean oil [40], or after 12–13 days for corn oil [41]. Panfrying or microwave heating of sea bream or sea bass with fresh commercial sunflower oil did not stimulate the formation of alkanals in fish [42], in accordance to our results. Hexanal was however found to be formed during the in vitro digestion of fresh commercial sunflower oil and this formation was especially evident during digestion of the oil previously heated for 4 days at 70 °C [43]. For future studies, it would therefore be interesting to study the oxidative stability during the culinary preparation and digestion of meat with commercial vegetable oils which were stored for a longer period of time.

In addition, the ratio of vegetable oil to meat varies widely among studies and could also explain discrepancies among studies. For instance, Martini et al. [16] found antioxidant effects of extra-virgin olive oil during digestion of turkey meat when added at a level of 2.5%, whereas in contrast, at higher added levels (5 and 10%), extra-virgin olive oil phenolic compounds increased lipid hydroperoxide formation from meat. When commercial sunflower oil (13.7%) was added to a diet containing red meat (17.1%), rats had increased plasma 4-HNE levels [14]. Compared to the latter study, the level of commercial vegetable oil added to beef in our study was much lower (3%). Therefore, more studies are warranted on the effects of varying oil to meat ratios, as was demonstrated for olive oil by Martini et al. [16].

## 5. Conclusions

In conclusion, cooking meat or fish with butter maintained the inherent n-6/n-3 PUFA ratio, as well as the lipid oxidative stability during digestion. The oxidizing potential of fish oil and safflower oil mixtures largely depended on the muscle matrix to which these was added. Especially in beef (digests), addition of safflower oil increased n-6 PUFA oxidation, whereas addition of fish oil increased n-3 PUFA oxidation without protective effect on n-6 PUFA oxidation. In contrast, cooking beef with 3% fresh commercial vegetable oils labeled as ‘suitable for heating’ did not promote oxidation during digestion in the present study.

## Data Availability

The data presented in this study are available on request.
